# Pro-inflammatory Stimulation of Monocytes by ANCA Is Linked to Changes in Cellular Metabolism

**DOI:** 10.3389/fmed.2020.00553

**Published:** 2020-09-08

**Authors:** Eóin C. O'Brien, Carla A. White, Jason Wyse, Emma Leacy, Richard K. Porter, Mark A. Little, Fionnuala B. Hickey

**Affiliations:** ^1^Department of Clinical Medicine, Trinity Health Kidney Centre, Trinity College Dublin, Dublin, Ireland; ^2^Discipline of Statistics and Information Systems, School of Computer Science and Statistics, Trinity College Dublin, Dublin, Ireland; ^3^School of Biochemistry and Immunology, Trinity Biomedical Sciences Institute (TBSI), Trinity College Dublin, Dublin, Ireland

**Keywords:** ANCA vasculitis, monocyte, immunometabolism, autoimmune, metabolism

## Abstract

Clinical and experimental data suggest that pathogenesis in anti-neutrophil cytoplasmic antibody (ANCA)-associated vasculitis is driven by ANCA-mediated activation of neutrophils and monocytes. While the role of neutrophils has been extensively investigated, the function of monocytes remains relatively understudied. We have previously demonstrated that stimulation of monocytes with anti-myeloperoxidase (MPO), but not anti-proteinase-3 (PR3), antibodies results in production of the pro-inflammatory cytokine IL-1β. Changes in cellular metabolism, particularly a switch to glycolysis, have recently been linked to activation of immune cells and production of IL-1β. Therefore, we investigated the metabolic profile of monocytes following ANCA stimulation. We found a significant increase in glucose uptake in anti-MPO stimulated monocytes. Interestingly, both anti-MPO and anti-PR3 stimulation resulted in an immediate increase in glycolysis, measured by Seahorse extracellular flux analysis. However, this increase in glycolysis was sustained (for up to 4 h) in anti-MPO- but not anti-PR3-treated cells. In addition, only anti-MPO-treated cells exhibited increased oxidative phosphorylation, a metabolic response that correlated with IL-1β production. These data indicate that monocyte metabolism is altered by ANCA, with divergent responses to anti-MPO and anti-PR3 antibodies. These metabolic changes may underlie pathologic immune activation in ANCA associated vasculitis, as well as potentially contributing to the differing clinical phenotype between PR3- and MPO-ANCA positive patients. These metabolic pathways may therefore be potential targets for therapeutic intervention.

## Introduction

Anti-neutrophil cytoplasmic antibody (ANCA) associated vasculitides (AAV) are a group of autoimmune conditions affecting the microvasculature (1). Most patients harbor autoantibodies directed against myeloperoxidase (MPO) or proteinase 3 (PR3). These antigens are found in the primary granules of neutrophils and lysosomes of monocytes and can be externalized when these cells are primed, thereby allowing engagement with circulating ANCA. The clinical phenotype of patients with anti-PR3 and anti-MPO antibodies is different, with the former often exhibiting granulomatous inflammation and the latter frequently having a sclerosing phenotype. Additionally, genomic analyses have demonstrated that AAV with PR3-ANCA is genetically distinct from that with MPO-ANCA (2). To date, our understanding of disease pathogenesis has been dominated by experimental work in neutrophils (3). However, recent data from our laboratory and others suggests a role for inflammatory monocytes in driving disease (4–6).

IL-1β is a key pro-inflammatory cytokine that induces both local and systemic inflammation through the production of cyclooxygenase type 2 (COX-2), nitric oxide, adhesion molecules, fever, and polarization of T cells [reviewed in (7)]. We previously described increased IL-1β production from human monocytes in response to stimulation with anti-MPO antibodies (4). This result has also been shown by other groups with IL-1β acting as a mediator of proinflammatory monocyte activation in a murine system (8).

Recent studies have shown that changes in intracellular metabolism play a major role in immune cell function and polarization (9). Rather than being secondary events, these changes have been shown to instruct cell function (10) and have been implicated in several autoimmune conditions (11, 12). The most frequent metabolic event in this setting involves a programmed switch from oxidative phosphorylation (oxphos) to aerobic glycolysis (13). Most work in this emerging “immunometabolism” field has focused upon macrophages and T cells. The production of IL-1β from murine bone marrow derived macrophages (BMDMs) is linked to a switch to aerobic glycolysis in response to pro-inflammatory signals including LPS (14, 15). Some studies have shown that, like macrophages, monocytes undergo a metabolic shift from oxphos to glycolysis in response to an acid environment induced by lactate accumulation (16). However, in experiments using HIV as the stimulus, the opposite effect was observed, with glycolysis being decreased in activated monocytes (17). Monocytes activated with HIV also display increased levels of the glucose transporter Glut-1 and have increased glucose uptake (18). These data show that the metabolic response of monocytes varies depending on stimulus and, therefore, focused analysis in monocytes using condition-specific stimuli is necessary.

Here, we have investigated ANCA-mediated metabolic changes in monocytes. We report divergent metabolic responses to anti-MPO and anti-PR3 stimulation. These differences correlate with the distinct inflammatory cytokine profiles which we have previously reported (4).

## Methods

### Isolation of CD14+ Monocytes

Peripheral blood mononuclear cells (PBMCs) were isolated by density gradient centrifugation using lymphoprep and monocytes were purified by positive selection. PBMCs were incubated with anti-CD14 magnetic beads (Miltenyi Biotec) as per the manufacturer's instructions and CD14+ cells were then isolated using an LS column.

### Stimulation of Monocytes for Measurement of 2-NBDG Uptake Analysis

CD14+ monocytes were plated at a density of 2 × 10^6^ cells/ml in Roswell Parks Memorial Institute (RPMI) Media supplemented with 10% fetal calf serum (FCS), 100 U/ml penicillin, 1 mg/ml streptomycin and 2 mM l-glutamine (cRPMI). Cells were treated with 5 μg/ml anti-MPO (clone 2C7), anti-PR3 (clone CLB-12.8), or isotype mAb for 60 min followed by incubation with 86.5 μg/ml 2-(N-(7-nitrobenz-2-oxa-1,3-diazol-4-yl)amino)-2-deoxyglucose (2-NBDG) for 60 min @ 37°C with 5% CO_2_. Conjugated anti-CD14 (clone RM02) antibody was added for the last 10 min of the incubation. Cells were then analyzed immediately on a Cyan ADP analyser (Beckman Coulter).

### Treatment of Monocytes With Inhibitors and Cell Death Assay

Monocytes were plated at a density of 2 × 10^6^ cells/ml. Cells were treated with 10 mM 2-deoxyglucose (2-DG), 8 μM oligomycin, 20 mM dichloroacetate (DCA), or MitoTempo and incubated for 20 min before the addition of 5 μg/ml anti-MPO mAb, isotype control or vehicle for 4 h @ 37°C with 5% CO_2_. IL-1β levels were assessed in supernatants by ELISA. Propidium iodide (PI) staining was performed in the appropriate experiments by addition of 10 μg/ml PI immediately prior to analysis on a Cyan ADP analyser (Beckman Coulter).

### Mitochondrial Stress Test and Glycolysis Stress Test

Monocytes were adhered to CellTak-coated 24-well plates at a concentration of 1 × 10^6^ per well as described above. Cells were stimulated with 5 μg/ml anti-MPO, anti-PR3, isotype or vehicle for 4 h at 37°C with 5% CO_2_. Mitochondrial stress test or glycolysis stress test were performed as per the manufacturer's instructions using a Seahorse XF24 analyser (Agilent). Sequential addition of oligomycin (2 μM), D-glucose (5.5 mM), carbonyl cyanide-p-trifluoromethoxyphenylhydrazone (FCCP) (4 μM), rotenone (4 μM), 2 deoxy-glucose (2-DG) (10 mM) allows for accurate calculation of mitochondrial respiratory capacity and glycolytic acidification. Each condition was evaluated in duplicate and a minimum of three measurements was performed following addition of each compound.

### Measurement of Real Time Metabolic Changes in Response to ANCA

Monocytes were adhered to Seahorse plates as described above. Anti-MPO, anti-PR3 or isotype antibodies were added to port A of an XFe24 FluxPak. For ECAR measurements, D-glucose was added to port B. For OCR measurements rotenone or vehicle was added to port B. The Cell plate was added to the Seahorse XFe24 analyser. Six initial basal measurements were performed followed by injection of port A. For ECAR experiments three more measurements were performed before injection of glucose followed by nine further measurements. For OCR experiments one measurement was performed before rotenone injection followed by nine further measurements.

### Measurement of Cellular and Mitochondrial ROS in Monocytes

Monocytes were isolated and stimulated with 5 μg/ml anti-MPO, anti-PR3 or isotype mAb or 5 ng/ml LPS for 1 h at 37°C with 5% CO_2_. For the final 30 min of the incubation cells were treated with either 5 μM CM-H_2_DCFDA (Thermo Fisher Scientific, Loughborough, UK), 2.5 μM MitoSOX red (Thermo Fisher Scientific, Loughborough, UK), or 10 μg/ml JC-1 (Thermo Fisher Scientific, Loughborough, UK). Anti-CD14 antibody was added for the final 10 min of the incubation. Cells were analyzed immediately on a Cyan ADP analyser.

### Study Approval

Buffy coat samples were obtained from the Irish Blood Transfusion Service, St. James's Hospital, Dublin with ethical approval from the School of Medicine Research Ethics Committee, Trinity College Dublin.

### Statistics

All statistics and correlations were performed using GraphPad Prism 6 software and nonparametric analyses were used for non-normal data. When comparing three or more groups, a one-wat ANOVA was performed for unpaired samples and a *post hoc* Friedman test was used for paired samples. Multiple comparisons were corrected using Dunn's multiple comparisons test. For comparisons between two groups, a students *T*-test was used. To assess the difference in across multiple parameters 2-way ANOVA with Tukey's *post hoc* multiple comparison test was used. Differences were only statistically significant (*p* < 0.05) when specified.

## Results

### Anti-MPO Stimulated Monocytes Produce IL-1β in a Glycolysis Dependent Manner

**Figure 1:**
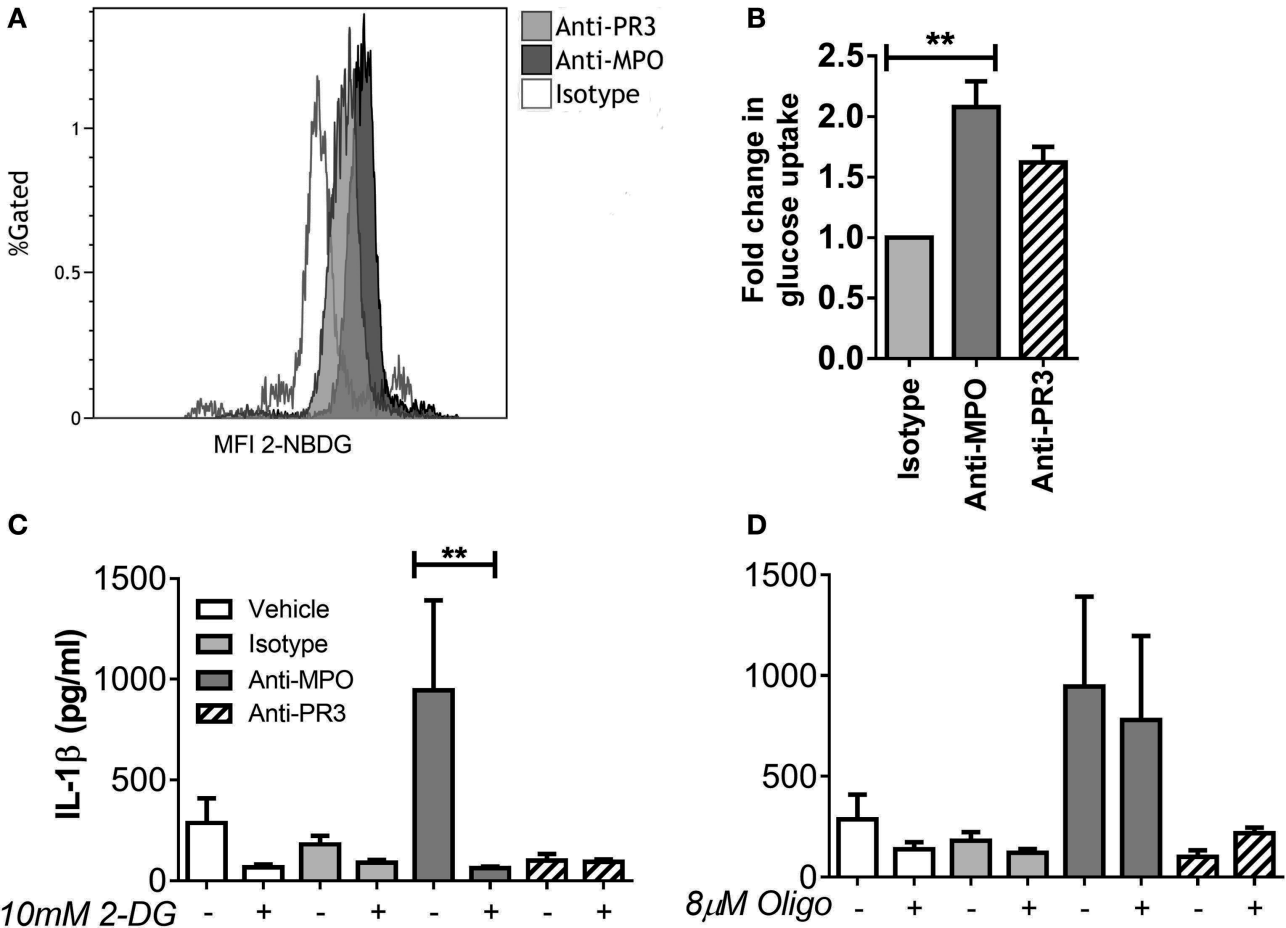
IL-1β production from anti-MPO stimulated monocytes requires glycolysis, but not oxphos. CD14+ monocytes were stimulated with mAb directed against MPO, PR3 or an isotype control, followed by incubation with 2-NBDG. Median fluorescence intensity (MFI) was compared between treatments. Representative overlays are shown for anti-MPO and anti-PR3 treatments **(A)**. Fold change in 2-NBDG uptake after antibody stimulation is shown for three independent donors **(B)**. CD14+ cells were incubated with either 10 mM 2-DG **(C)** or 8 μM oligomycin **(D)** and then stimulated with 5 μg/ml mAb directed against MPO, PR3 or isotype control antibody. IL-1β was measured in supernatants by ELISA. Data are presented as the mean and SEM. Statistical analysis was performed by students *T*-test **(B)** (**p < 0.01) or one-way ANOVA with Friedman's post-test **(C,D)** (**p < 0.01) (*n* = 6).

We have previously demonstrated differential cytokine production in monocytes in response to anti-MPO and -PR3, with anti-MPO, but not anti-PR3 leading to increased secretion of IL-6, IL-8, and IL-1β (4). As IL-1β production is linked to changes in intracellular metabolism, we sought to investigate the metabolic correlates of IL-1β production by anti-MPO and anti-PR3 stimulated monocytes. Firstly, we investigated the overall glucose uptake by monocytes in response to these stimuli by measuring uptake of 2-[N-(7-nitrobenz-2-oxa-1,3-diazol-4-yl) amino]-2-deoxy-D-glucose (2-NBDG), a non-metabolisable fluorescent analog of glucose. This molecule is taken up in the same way as glucose but is not broken down by the cell and it's fluorescence can therefore be used as a surrogate marker for glucose uptake (19). As expected, based on its ability to induce IL-1β, anti-MPO stimulation resulted in significantly enhanced glucose uptake (Figures 1A,B). We therefore studied the role of glucose metabolism in anti-MPO and -PR3 antibody induced production of this cytokine. We used 2-deoxyglucose (2-DG) to block hexokinase, and therefore inhibit glycolysis, and oligomycin to block the electron transport chain, and thus oxphos. 2-DG treatment of monocytes abolished anti-MPO induced IL-1β production (without causing cellular injury as measured by PI exclusion, (Supplementary Figure 1), indicating that glycolysis is required for IL-1β production in these cells (Figure 1C). Conversely, oligomycin treatment had no effect on anti-MPO-induced IL-1β production (Figure 1D).

### Anti-MPO Stimulation of Monocytes Results in Both Increased Oxygen Consumption and Glycolysis

**Figure 2:**
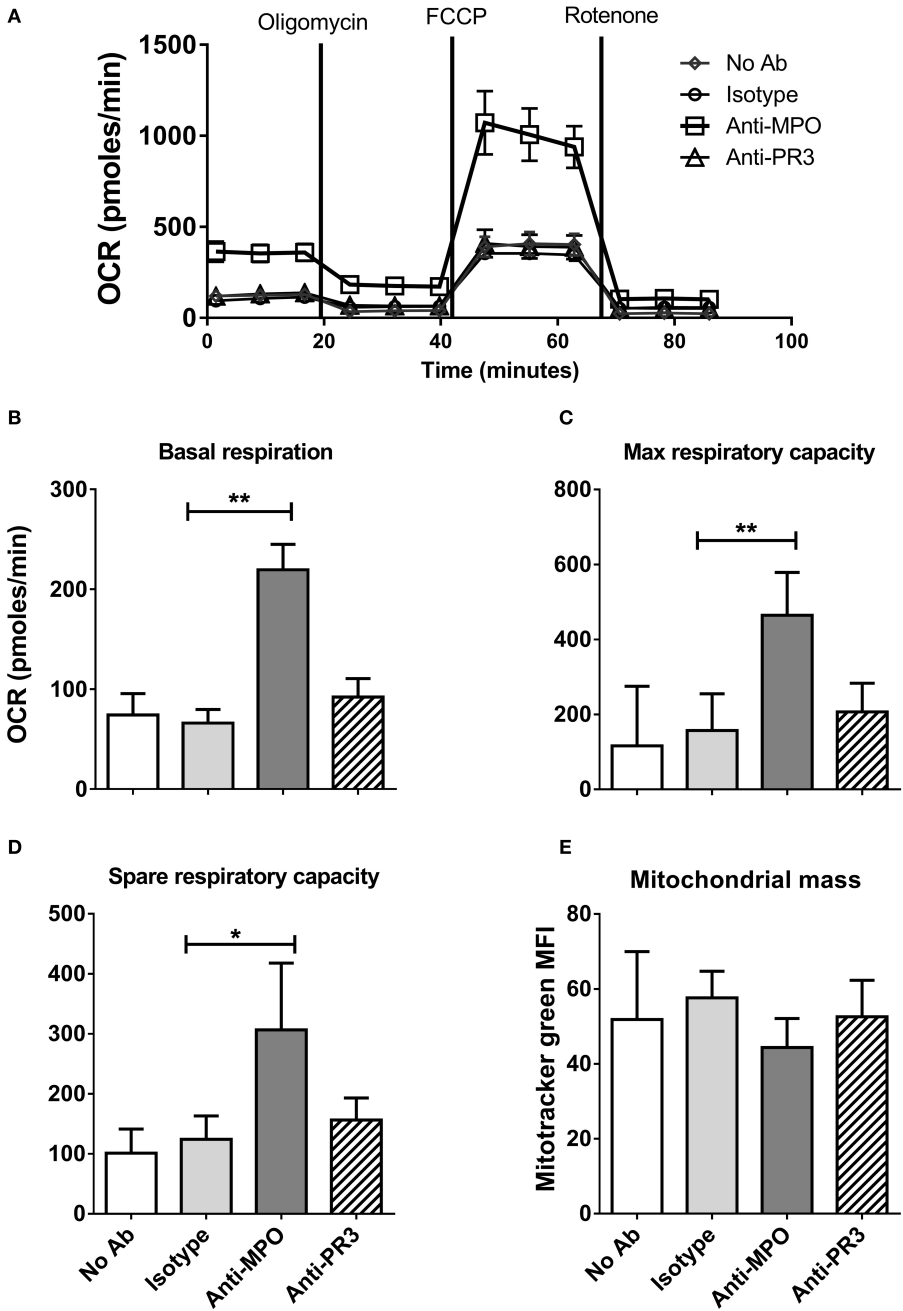
Anti-MPO, but not anti-PR3 stimulation of monocytes leads to increased oxidative respiration and respiratory capacity. CD14+ monocytes were stimulated with 5 μg/ml mAb directed against MPO, PR3 or isotype control antibody. Initial oxygen consumption rate (OCR) was measured followed by addition of 4 μM oligomycin, 2 μM FCCP and 4 μM rotenone **(A)**. Basal respiration levels were calculated by subtracting the non-mitochondrial respiration (post rotenone addition) from the initial OCR readings **(B)**. Maximum respiratory capacity was calculated by subtracting non-mitochondrial respiration from OCR values following the addition of FCCP **(C)**. Spare respiratory capacity was calculated by subtracting basal from maximum respiration **(D)**. Mitochondrial mass was measured by flow cytometry of cells incubated with 50 nM MitoTracker Green following stimulation as above **(E)**. Data are presented as the mean and SEM. Statistical analysis was performed by one-way ANOVA with Friedman's post-test (*p < 0.05, **p < 0.01) (**A–D** *n* = 6, **E** *n* = 3).

To further define the changes in monocyte metabolism in response to anti-MPO and -PR3 antibody stimulation we performed Seahorse Extracellular Flux analysis, which permits simultaneous measurement of both extracellular acidification rate (ECAR) and oxygen consumption rate (OCR), markers of glycolysis and oxphos, respectively. We hypothesized that the increase in glucose uptake in response to anti-MPO would be associated with a switch to aerobic glycolysis, with a corresponding decrease in OCR. However, we found that anti-MPO treatment markedly increased monocyte basal OCR (Figures 2A,B), as well as increasing maximum and spare respiratory capacity (Figures 2C,D). This increase was not found in anti-PR3 stimulated cells (Figures 2A–D). The increase in maximum respiratory capacity in response to anti-MPO stimulation is particularly interesting due to the relatively short stimulation time (4 h). The primary mechanism by which respiratory capacity is increased is via mitochondrial biogenesis. However, an increase in mitochondrial mass over our 4 h stimulation is unlikely. Using mitotracker green we confirmed that our anti-MPO stimulation had no effect on mitochondrial mass (Figure 2E), suggesting that the observed upregulated oxygen consumption is not due to increased mitochondrial biogenesis.

**Figure 3:**
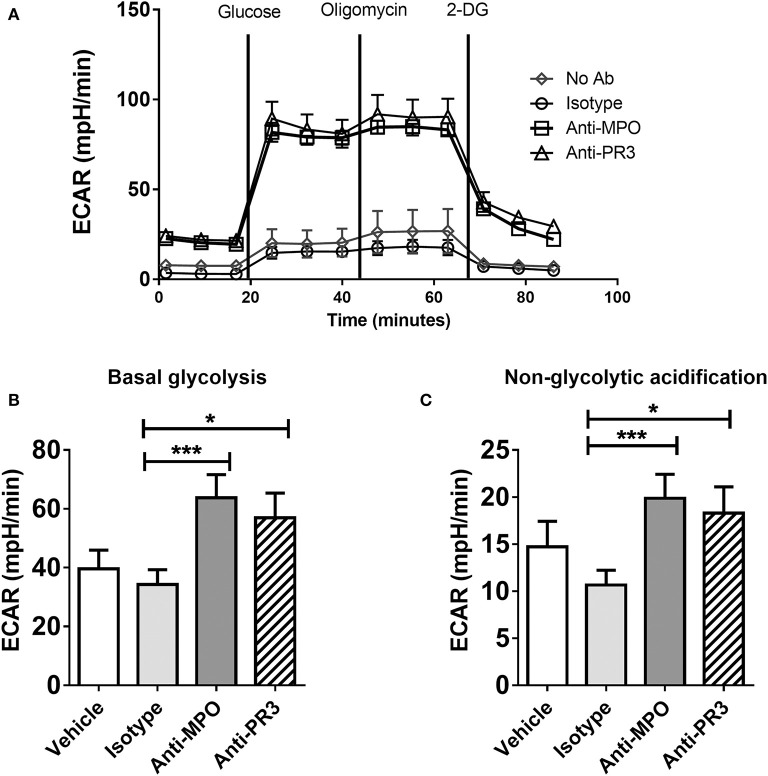
Anti-MPO and anti-PR3 stimulation results in an upregulation of glycolysis in monocytes. CD14+ monocytes were stimulated with 5 μg/ml mAb directed against MPO, PR3 or isotype control antibody. Extracellular acidification rate (ECAR) was measured at basal levels and following the addition of 4.5 mM D-glucose, 4 μM oligomycin and 10 mM 2-DG **(A)**. Glycolytic rate was calculated by subtracting non-glycolytic acidification (post 2DG treatment) from the rate of acidification following the addition of glucose **(B)** Non-glycolytic acidification was defined as the ECAR rate after 2-DG addition **(C)**. Data are presented as the mean and SEM. Statistical analysis was performed by one-way ANOVA with Friedman's post-test (*p < 0.05, ***p < 0.001) (*n* = 11).

In contrast to OCR, which was increased only by anti-MPO antibody, stimulation with both anti-MPO and -PR3 increased basal glycolysis and non-glycolytic acidification (as determined by ECAR) (Figures 3A–C). Contrary to the expected switch to aerobic glycolysis, we instead observed parallel increases in glucose uptake, OCR and ECAR in response to anti-MPO, suggesting a broad up-regulation of monocyte bioenergetics.

### Changes in Monocyte Cellular Metabolism in Response to ANCA Occur Immediately After Stimulation

**Figure 4:**
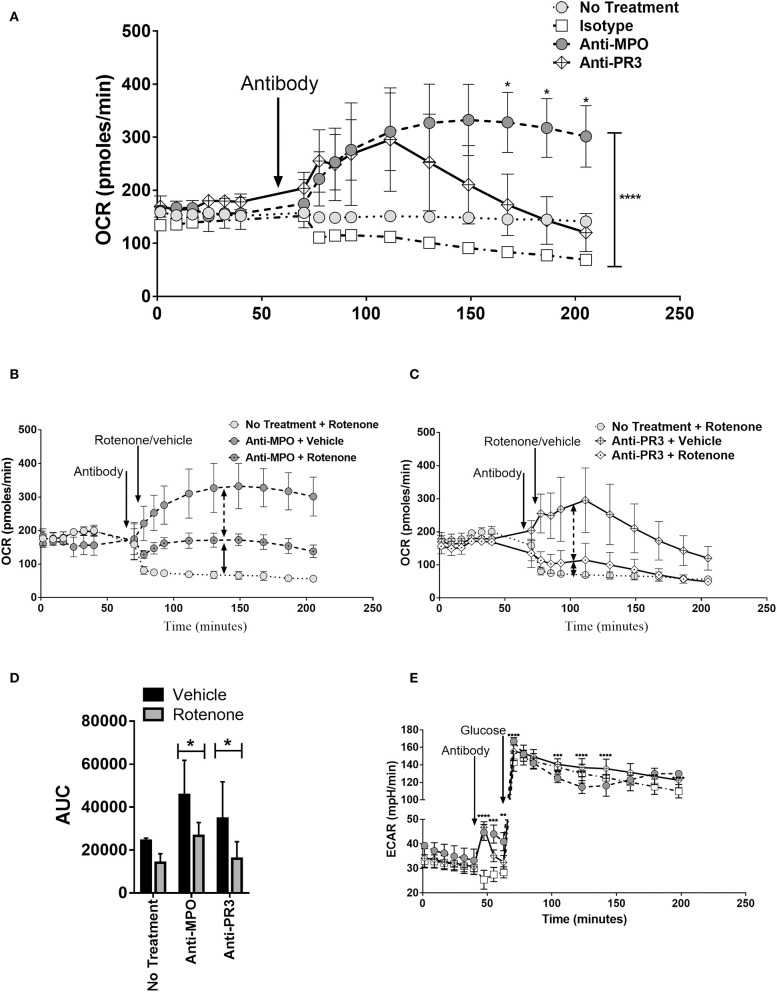
Anti-MPO and anti-PR3 stimulated monocytes have differing OCR and ECAR kinetic patterns. OCR of CD14+ monocytes was measured at regular intervals following the addition of 5 μg/ml mAb directed against MPO **(A,B)**, PR3 **(A,C)** or isotype control antibody **(A–C)** by Seahorse extracellular flux analysis. Cells were subsequently treated with rotenone (black dot in symbol) or vehicle **(B,C)**. The area under the curve (AUC) from the point of antibody injection was calculated **(D)**. ECAR was measured at regular intervals following the addition of 5 μg/ml mAb directed against MPO **(A,B)**, PR3 **(A,C)** or isotype control antibody **(A–C)** by Seahorse extracellular flux analysis **(E)**. Statistical analysis was performed by two-way ANOVA with Tukey's multiple comparisons test (*p < 0.05, **p < 0.01, ***p < 0.001, ****p < 0.0001) (*n* = 3). Light gray circles, no treatment; white boxes, isotype control; dark gray circles, anti-MPO; white diamond, anti-PR3.

The changes in monocyte metabolism in response to ANCA described above occurred within 4 h of stimulation. The relatively short timeframe of the glycolytic and oxphos responses led us to investigate the real-time kinetics, using the Seahorse analyser, of these responses by adding anti-MPO and anti-PR3 directly to the cells. Changes in OCR (Figure 4A) were measured in real-time immediately following addition of antibody. Both anti-MPO and -PR3 stimulation resulted in immediate increases in OCR of similar magnitude. Approximately 45 min after antibody stimulation, OCR levels in anti-PR3 stimulated monocytes reduced rapidly to basal levels, whereas the OCR in anti-MPO stimulated cells continued to rise and was maintained for the duration of the experiment (Figure 4A). Such an immediate increase in oxygen consumption raised the possibility that it was due to non-mitochondrial oxygen consumption, such as through reactive oxygen species production by nicotinamide adenine dinucleotide phosphate (NADPH) oxidase. We addressed this question using the oxphos inhibitor rotenone, which specifically inhibits mitochondrial oxygen consumption. Rotenone reduced overall OCR following both anti-MPO (Figure 4B) and anti-PR3 (Figure 4C) monocyte stimulation, although not completely to control levels, thereby confirming that much of the observed oxygen consumption was due to oxphos, with a small contribution from NADPH oxidase (Figure 4D). Interestingly, we found an immediate increase in ECAR in the absence of glucose following addition of both antibodies, indicating an increase in non-glycolytic acidification (Figure 4E). Upon glucose addition, ECAR increased in response to both antibodies by a similar amount. This increase was followed by a rapid decline in anti-MPO treated cells followed by a subsequent increase by 4 h post glucose addition while anti-PR3 stimulated cells showed a steady decline from their peak after glucose injection (Figure 4E).

### Mitochondrial Reactive Oxygen Species Induced by Anti-MPO Stimulation Are Required for IL-1β Production

**Figure 5:**
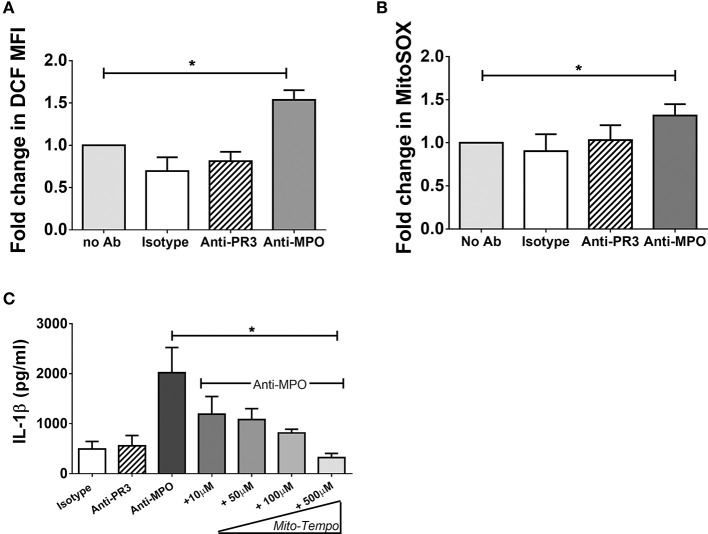
Anti-MPO induced IL-1β production is abrogated by the mitochondrial ROS scavengers MitoTempo. CD14+ monocytes were incubated with increasing concentrations of MitoTempo followed by stimulation with 5 μg/ml mAb directed against MPO, PR3, or isotype control antibody. Supernatants were removed and IL-1β was measured by ELISA **(A)**. For flow cytometry experiments, monocytes were stimulated as above. CM-H_2_DCFDA **(B)** or MitoSOX Red **(C)** was then added to the cells **(B)** and the MFI was determined by flow cytometry. Statistical analysis was performed by one-way ANOVA with Friedman's post-test (*p < 0.05) (*n* = 5).

We have found that monocytes stimulated with anti-MPO antibodies increase oxphos in parallel with glycolysis, but only inhibition of glycolysis blocks IL-1β production (Figure 1). The increased oxidative metabolism may be required for the production of biosynthetic precursors or to generate ROS. We tested the hypothesis that mitochondrial ROS (mROS) contribute to the pro-inflammatory effect of anti-MPO antibodies on monocytes. Using the general oxidative stress indicator CM-H_2_DCFDA we detected an increase in cellular ROS in anti-MPO treated cells (Figure 5A). Similar experiments employing the mROS-specific probe MitoSOX also indicated an increase in mROS in response to anti-MPO (Figure 5B). In order to examine the importance of this increase in the inflammatory activation of monocytes by anti-MPO we pre-treated cells with the mROS-specific scavenger MitoTempo. We found that the IL-1β produced by monocytes in response to anti-MPO was inhibited by MitoTempo in a dose-dependent manner (Figure 5C), indicating a role for mROS in this pro-inflammatory pathway. ANCA stimulation did not alter monocyte mitochondrial membrane potential as measured by JC-1 staining (Supplementary Figure 2).

## Discussion

The fundamental importance of metabolic changes in immune cell activation is becoming a key area of interest. Cellular metabolic pathways are complex and often interconnected, with multiple pathways changing in a particular cell type in response to a specific stimulus (20). Macrophages have become the subject of intense study in terms of their metabolic profile in response to pro-inflammatory stimuli such as LPS. These studies have predominantly focused on murine bone marrow derived macrophages (BMDMs) and tissue resident macrophages (10, 21, 22) and have largely neglected blood-borne myeloid lineage cells, specifically monocytes. While monocytes can translocate to tissues and differentiate into macrophages or dendritic cells, they also have effects while still in the circulation (23, 24). These functions may be particularly important in diseases where the mechanism of action is not localized to a specific tissue, but rather to the vasculature, as is the case in AAV, and where the autoantigens are located within the monocyte.

18-F-Fluorodeoxyglucose Positron Emission Tomography with Computed Tomography (PET) which quantifies glucose uptake, has shown increased rates of glucose uptake in the affected organs of patients with AAV (25), indicating probable upregulation of cellular metabolism in these immune cell rich areas. Here, we have shown that ANCA treatment of monocytes results in increased glucose uptake as measured by the fluorescent glucose analog 2-NBDG. This compound has been shown to be a useful measure of glucose uptake (19) although some recent studies suggest that uptake of this glucose analog may be lower than that of radiolabelled glucose (26). As we found increased 2-NBDG uptake in response to both anti-MPO and anti-PR3 stimulation, any artifactual diminished uptake of this compound implies an even greater increase in glucose uptake in monocytes in response to ANCA.

We investigated the hypothesis that altered cellular metabolism, in response to increased glucose uptake, is involved in the response of monocytes to ANCA stimulation. The current paradigm in pro-inflammatory immune cell activation is that these cells shift to a more glycolytic phenotype. While this is the case in most cell types, it is also true that some plasticity between pathways exists in cells depending on their level of activation. Treg cells for example, require oxphos for long term survival (27) but exhibit enhanced glycolysis in the initial stages of activation (28). Macrophages have increased reliance on specific metabolic pathways, depending on their polarization. Contrary to this, we have demonstrated that ANCA treatment of monocytes results in broad upregulation of glucose metabolism. This may be the result of monocytes carrying out pro-inflammatory effector functions while also undergoing the process of differentiation into the macrophages found in lesions of AAV patients (29).

One of the most interesting aspects of this study are the similarities and differences in response to anti-MPO and anti-PR3 stimulation. In our earlier work, we showed that only anti-MPO treatment resulted in IL-1β production. Here we show that the initial metabolic response to both antibodies is remarkably similar with both glycolysis and oxphos being increased. However, only anti-MPO treatment resulted in sustained oxphos upregulation and this, along with increased mROS, correlated with the pro-inflammatory IL-1β production. Some of the metabolic effects of ANCA on monocytes mimic those seen in pro-inflammatory macrophages. In both cases, blocking glycolysis or mitochondrial ROS (mROS) results in a downregulation of the secretion of proinflammatory cytokines (15). In LPS stimulated macrophages, which have switched to predominantly glycolytic metabolism, mROS are produced through complex I (30). This mROS production has been shown to be dependent on an increase in mitochondrial membrane potential (15). It has been hypothesized that one reason for the switch to glycolysis in activated macrophages is the need to maintain mitochondrial membrane potential to allow for activation of this pathway (31). In response to TCR activation, some activated T cells also increase both glycolysis and oxidative respiration upon activation (32–34) and it is thought that the oxidative respiration may be needed in order to produce ROS (35). Our findings, that inhibition of glycolysis and mROS abrogate IL-1β production, while oligomycin had no effect, suggest that monocytes following ANCA stimulation may require glycolysis feeding into oxphos in order to produce ROS, rather than altering membrane potential to drive these pro-inflammatory pathways. ANCA activated monocytes may therefore be more similar to T cells than macrophages in this regard.

Patients with anti-PR3 and anti-MPO often display differing disease phenotypes with anti-PR3 disease being particularly associated with granuloma formation. The differences between the overall metabolic phenotype of PR3 vs. MPO stimulated cells may be an important factor in these cells forming granulomas. Granuloma formation has been shown to be reliant on glycolytic metabolism in Mycobacterium Tuberculosis infection (36). The upregulation of these pathways alone in anti-PR3 stimulated monocytes may therefore play a role in granuloma formation in PR3-ANCA vasculitis. The upregulation of both oxidative phosphorylation and glycolysis seen in anti-MPO treated monocytes indicates a more pro-inflammatory phenotype. This suggests that these cells may be having increased effector functions to both recruit other immune cells and to directly damage tissue. This difference is emphasized by the increase in pro-inflammatory cytokines from these cells along with their increased ROS production. Both IL-1β and ROS have been shown to result in tissue damage which may help drive the glomerulonephritis that is observed in some patients with MPO-ANCA vasculitis (37).

How the extent of metabolic changes correlates with the role of monocytes in PR3 and MPO ANCA vasculitis is not known. We hypothesize that the short kinetics of anti-PR3 stimulated metabolic changes in monocytes prevents these cells from effectively upregulating ox phos and allows them to remain glycolytic without enhancing their inflammatory cytokine production. This in turn provides the potential for these cells to form granulomas associated with PR3 ANCA vasculitis. In contrast, the prolonged metabolic changes seen in MPO treated monocytes results in profound a pro-inflammatory phenotype which may result in tissue damage often found in patients with MPO-ANCA vasculitis.

This study has provided initial data into the response of these pathways to ANCA. We have shown how ANCA stimulation leads to shifts in monocyte cellular metabolism and how anti-MPO and anti-PR3 antibodies have differing effects on the metabolism of these cells. The links we have shown between pro-inflammatory cytokine production in anti-MPO treated monocytes and these changes in metabolism suggest that the overall differences between anti-MPO and anti-PR3 treated cells, and clinical phenotypes, may be at least partially explained by altered metabolic phenotypes. These alterations in metabolism may provide future targets for clinical interventions in an antibody type stratified treatment approach.

## Data Availability Statement

The raw data supporting the conclusions of this article will be made available by the authors, without undue reservation.

## Ethics Statement

The studies involving human participants were reviewed and approved by School of Medicine Research Ethics Committee Trinity College Dublin. Written informed consent for participation was not required for this study in accordance with the national legislation and the institutional requirements.

## Author Contributions

EO'B designed and performed experiments, analyzed data, and wrote the manuscript. CW and EL performed experiments. JW performed statistical analysis. RP conceptualized experiments and edited the manuscript. ML and FH designed experiments, analyzed data, and wrote the manuscript. All authors contributed to the article and approved the submitted version.

## Conflict of Interest

The authors declare that the research was conducted in the absence of any commercial or financial relationships that could be construed as a potential conflict of interest.
