# Supplementary material for: Pro-inflammatory Stimulation of Monocytes by ANCA Is Linked to Changes in Cellular Metabolism
Source: Front Med (Lausanne). 2020 Sep 8;7:553. doi: 10.3389/fmed.2020.00553 (PMC7509421; doi:10.3389/fmed.2020.00553)
Supplement: Supplementary file 1 [file Data_Sheet_1.PDF]

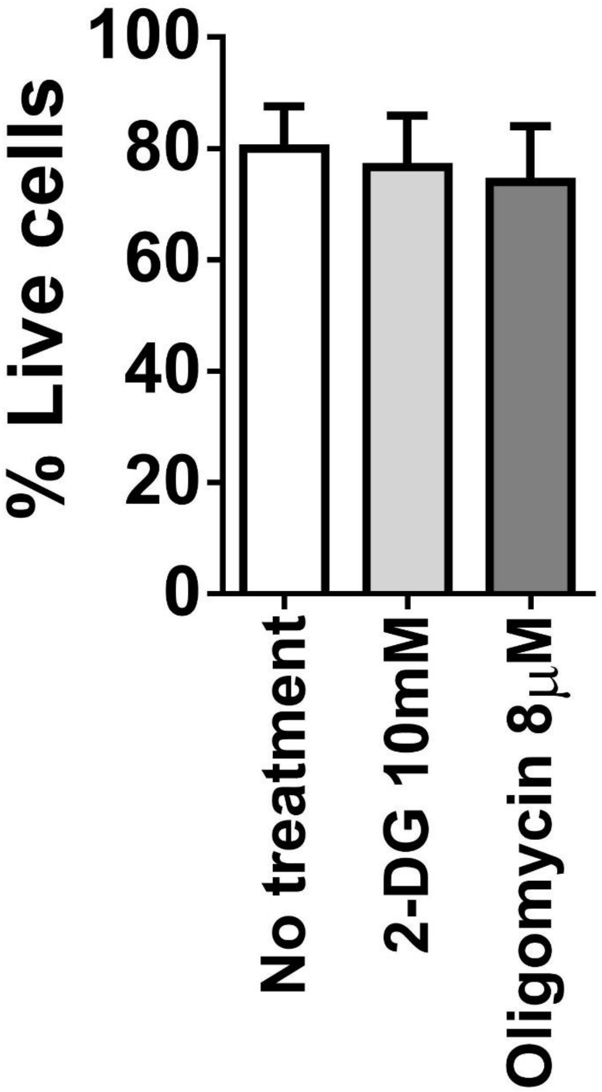

Supplementary figure 1. Concentrations of 2DG and oligomycin used were not toxic to CD14+ monocytes. CD14+ monocytes were isolated from healthy control PBMCs using MACS separation. CD14+ cells were incubated with either 10mM 2-DG or 8µM oligomycin and then stimulated with 5µg/ml mAb directed against MPO, PR3 or isotype control antibody. Live cells were quantified by flow cytometry using propidium iodide exclusion.

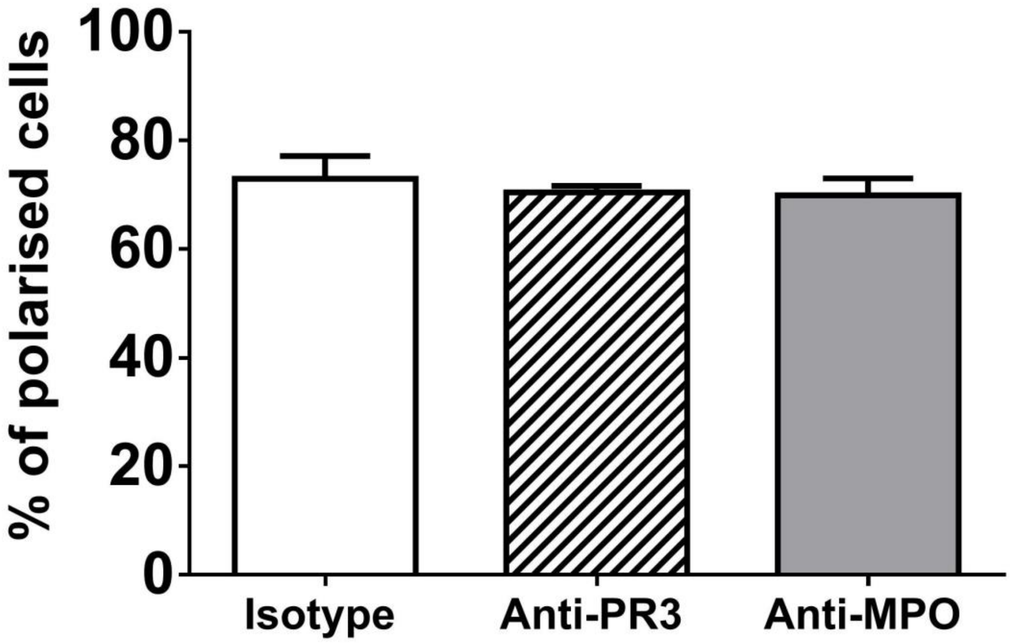

Supplementary figure 2. ANCA stimulation does not disrupt the mitochondrial membrane potential of CD14+ monocytes. CD14+ monocytes were isolated from the PBMCs of healthy volunteers by MACS separation. Cells were stimulated with 5µg/ml mAb directed against MPO, PR3, or isotype control antibody followed by incubation with JC-1 and the proportion of cells moving from green fluorescence to red fluorescence was determined by flow cytometry. (n=3)
